# The Surgeon and the Algorithm: Why the Vatican’s Bioethical Blueprint Is Essential for Modern Surgical Practice

**DOI:** 10.7759/cureus.111430

**Published:** 2026-06-24

**Authors:** Andrew Lourdunathan

**Affiliations:** 1 General Surgery, St. Martha's Hospital, Bengaluru, IND; 2 General Surgery, Shri Sathya Sai Medical College and Research Institute, Chennai, IND

**Keywords:** artificial intelligence in surgery, bioethics, digital sovereignty, health data colonialism, informed consent, machine learning, medical theology, minimal access surgery, patient advocacy, surgical ethics

## Abstract

The rapid integration of artificial intelligence (AI), intraoperative predictive analytics, and automated tracking platforms within modern surgical workflows presents emerging ethical challenges regarding patient autonomy, transparency, and data governance. This editorial explores how the recent Papal Encyclical *Magnifica Humanitas* provides a human-centered bioethical blueprint for contemporary surgical practice. By emphasizing the intrinsic dignity of the patient, this framework offers guidance against reducing individuals to standardized data metrics. The essay further examines concerns surrounding "health data colonialism" - the extraction and commercialization of localized surgical registries, clinical video logs, and patient data by proprietary models without adequate patient transparency. To address these ethical challenges, we propose an overhaul of standard informed consent protocols to include a "Digital Sovereignty Addendum." True patient advocacy in the digital age requires transparently informing patients whether their clinical footprints are utilized for algorithmic training, ensuring that technological advancement remains aligned with human-centered clinical care.

## Editorial

The need for a new ethical compass

The integration of artificial intelligence (AI), predictive analytics, and automated data platforms into modern surgical practice has created unprecedented opportunities to improve clinical decision-making, efficiency, and patient outcomes [1]. At the same time, these technologies raise complex questions regarding autonomy, accountability, transparency, and the preservation of human dignity [2]. Existing bioethical frameworks, including principle-based ethics and human rights approaches, continue to provide valuable guidance; however, emerging challenges surrounding algorithmic decision support, secondary use of patient data, and digital governance have generated renewed ethical debate.

In this context, the recent Papal Encyclical *Magnifica Humanitas* [3] offers a distinctive contribution to contemporary discussions on surgical ethics. Rather than rejecting technological innovation, the document emphasizes the inherent dignity of the human person and argues that technological advancement should remain accountable to human flourishing. This editorial examines how these principles may inform current debates surrounding AI-assisted surgery, digital consent, and data sovereignty, while acknowledging the broader plurality of ethical perspectives that shape modern healthcare.

The bioethical core: the "patient face" versus the "Babel syndrome"

A primary danger in high-tech medicine is what the encyclical calls the "technocratic paradigm" - the tendency to let the logic of efficiency, control, and profit dictate clinical decisions. In surgical practice, this manifests as the temptation to reduce the profound mystery of the human person into standardized performance metrics, analytics, and data streams [1].

The Vatican rightly points out that while AI can process vast amounts of data at incredible speeds, it is fundamentally devoid of a moral conscience. Algorithmic systems do not undergo real experiences, nor do they understand compassion, mercy, or the deeper existential meaning of healing. When we allow automated systems to dictate patient pathways or determine resource allocation without strict human oversight, we risk falling into the "Babel syndrome," where human dignity is sacrificed for mechanical efficiency.

The theological advice from *Magnifica Humanitas *is an essential corrective for the modern clinician: the quality of our care must always be measured by our ability to recognize the patient as an irreplaceable face, not merely a function [3]. AI should be utilized strictly as an assistant that reduces administrative friction, ultimately giving the surgeon more time to look the patient in the eye, honor their existential dignity, and practice genuine, human-centered care [1].

Health data colonialism: the new frontier of bioethics

International sharing of health data has contributed substantially to scientific discovery, quality improvement initiatives, and the development of safer and more effective AI systems. Diverse datasets can improve algorithmic performance, reduce bias, and facilitate advances in patient care across geographic boundaries. Nevertheless, ethical concerns arise when data collection, governance, and commercialization occur without meaningful transparency, equitable benefit-sharing, or adequate patient understanding. It is within this context that concerns regarding health data colonialism have emerged.

Perhaps the most urgent bioethical issue faced today revolves around patient data privacy and ownership [4]. AI models are trained on massive datasets, but in the surgical world, that "data" consists of the deeply personal biological, clinical, and anatomical information of patients [5].

*Magnifica Humanitas* delivers a striking and necessary warning about a new form of resource extraction: health data colonialism. Today, transnational tech companies and private entities are harvesting health data, epidemiological profiles, and genetic maps, treating them as the new "rare earths" of global power. This data is routinely used to build proprietary predictive models, often without the explicit understanding or structural benefit of the populations who provided it [4].

From a bioethical standpoint, the Vatican's assertion that this data must be treated as a common good rather than an instrument of asymmetric dominance is absolutely correct. If we ignore this warning, the digital age will simply become a new, unchecked form of colonial exploitation under a technical guise [4]. This theological stance demands a radical, immediate update to current informed consent protocols [5].

Overhauling surgical consent for the digital age

Currently, standard surgical consent forms are primarily focused on the physical risks of a procedure, such as bleeding, infection, or damage to adjacent structures. However, the Vatican’s ethical framework insists that patient autonomy and transparency must be fiercely protected in the digital realm as well. To align practice with the principles of social justice and the universal destination of goods, informed consent must be expanded to encompass the patient's digital footprint.

True patient advocacy requires that standard surgical consent forms be modified to address the following issues [5].

Algorithmic Transparency

Patients must be explicitly informed if their surgical video logs, clinical outcomes, or biological data will be utilized to train proprietary AI models or optimize robotic software [1,5].

Data Sovereignty

Consent protocols must clearly outline where the patient’s digital data will be stored, who controls the underlying models, and whether it will be monetized or transferred to private third parties [4,5].

The Right to Decide

Clinicians must restore to patients the definitive ability to decide how their most intimate clinical data is utilized, ensuring they are treated as an end in themselves and never as a mere commodity to be sold off [5].

To align our practice with the principles of social justice and the universal destination of goods, informed consent must be expanded to encompass the patient's digital footprint.

The proposed Digital Sovereignty Consent Addendum should be regarded as a conceptual framework intended to stimulate discussion among clinicians, ethicists, institutions, and policymakers. It is not presented as a finalized legal or regulatory instrument but rather as a practical example of how existing informed consent processes might evolve to address emerging questions regarding algorithmic transparency, secondary data use, and patient autonomy in the digital age. A practical framework for this intervention is provided in the Appendix.

Limitations

This editorial is primarily a normative and conceptual analysis rather than an empirical investigation. Consequently, the arguments presented are not supported by prospective clinical outcome data, patient-reported measures, or formal assessments of clinician attitudes toward algorithmic governance. Future studies should evaluate whether the proposed principles influence patient trust, informed consent quality, shared decision-making, or clinical outcomes.

Furthermore, although *Magnifica Humanitas *provides a coherent ethical framework grounded in Catholic social teaching, alternative secular, human rights-based, and pluralistic bioethical traditions may arrive at similar conclusions regarding patient autonomy, transparency, accountability, and protection from exploitation. The applicability of this framework may therefore vary across healthcare systems, cultural settings, and legal environments [2].

The discussion of health data colonialism intentionally highlights potential risks associated with unequal data governance and commercialization of health information. However, international data sharing has also generated substantial scientific, educational, and public health benefits. Future scholarship should focus on identifying governance structures that preserve innovation while ensuring transparency, fairness, and equitable distribution of benefits.

Finally, the rapid evolution of AI technologies, privacy regulations, and data governance frameworks may alter contemporary understandings of algorithmic transparency, data ownership, and digital sovereignty. As a result, the ethical recommendations proposed in this editorial should be viewed as adaptive principles requiring periodic reassessment as technology and regulatory environments continue to evolve [4,5].

Conclusion: embracing the theological standard

As the boundaries of surgical innovation continue to expand through robotics, machine learning, and advanced digital platforms, clinicians must ensure that technological progress remains aligned with the fundamental purpose of medicine: the care of persons. Questions surrounding algorithmic influence, data governance, and informed consent are no longer theoretical concerns but practical challenges that increasingly affect everyday surgical practice [1].

Regardless of one's religious or philosophical commitments, the emergence of surgical AI necessitates renewed attention to patient dignity, transparency, accountability, and meaningful informed consent. *Magnifica Humanitas* contributes a distinctive perspective to this discussion by emphasizing that technological innovation should remain accountable to the human person whom it is intended to serve [3]. By fostering dialogue on digital consent and responsible stewardship of patient data, the surgical community can help ensure that technological advancement enhances, rather than diminishes, the humanity at the center of clinical care.

Appendix

Proposed Informed Consent Addendum for Digital Sovereignty in Surgery

**Figure 1:**
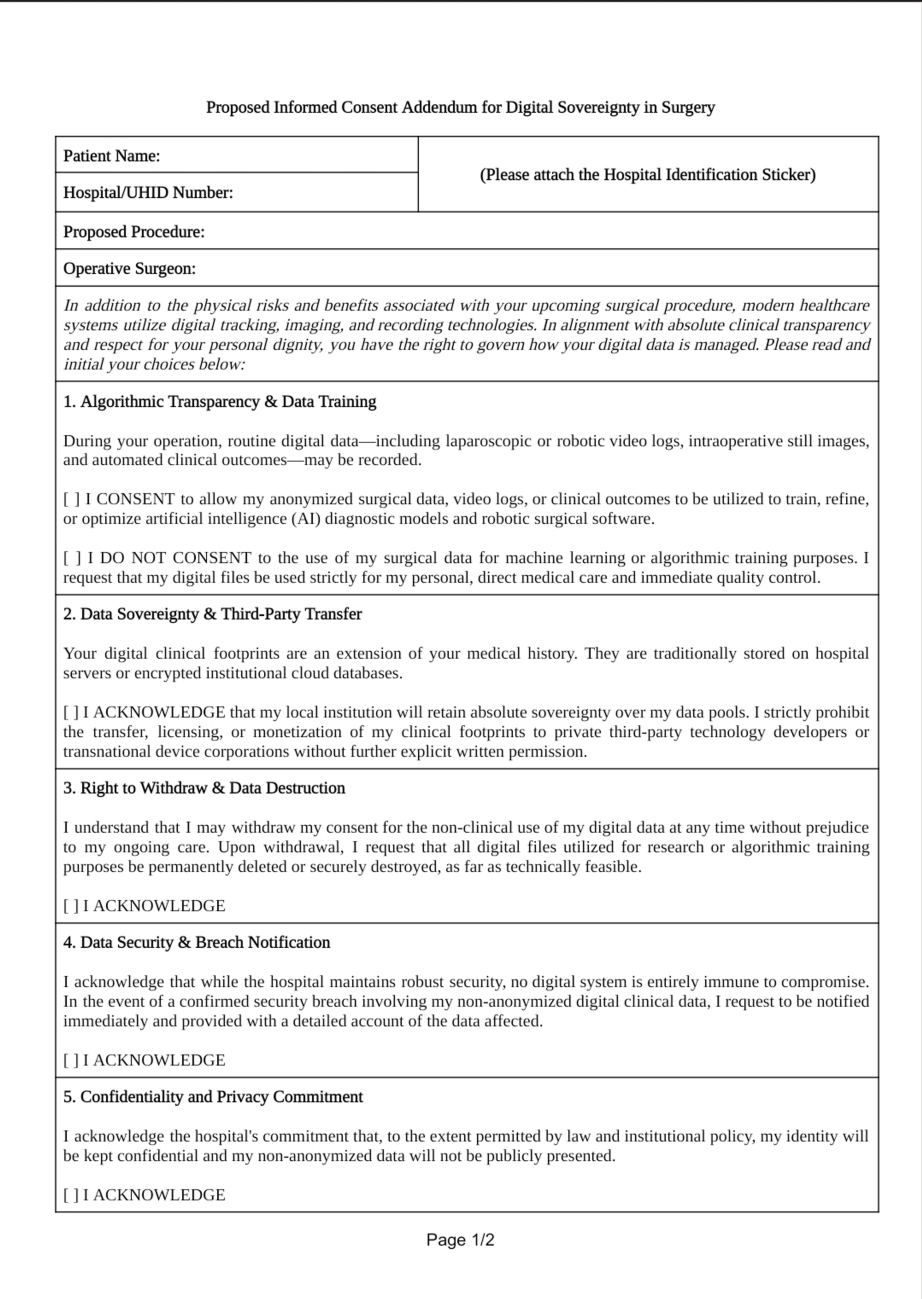
Digital Consent Addendum, page 1

**Figure 2:**
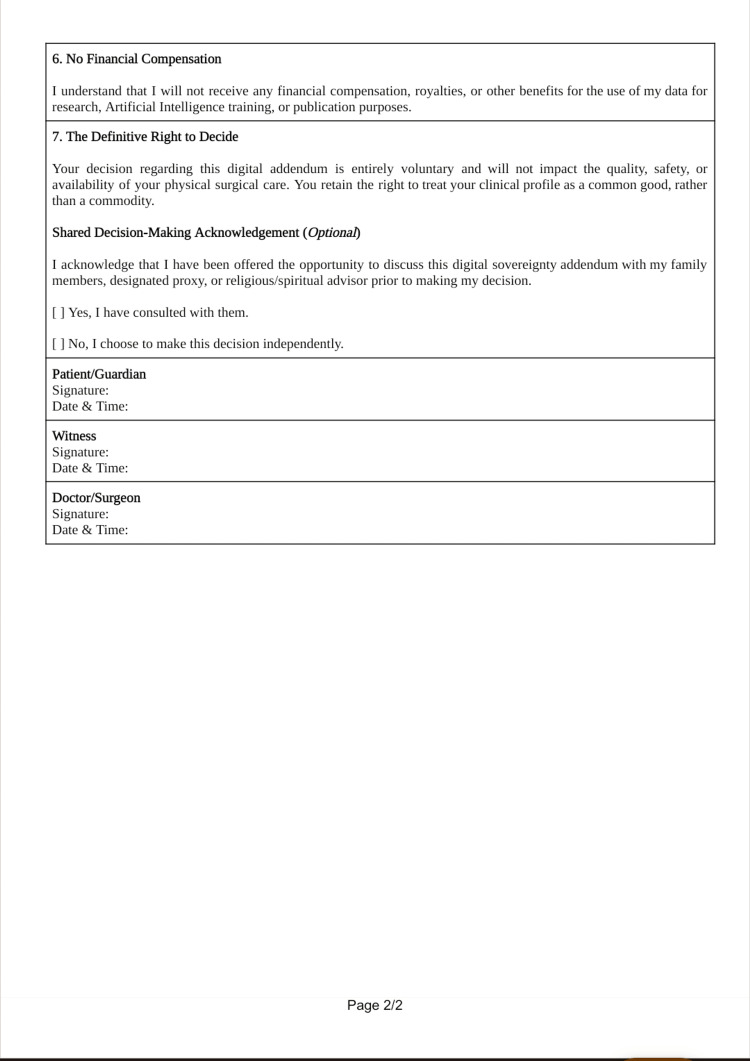
Digital Consent Addendum, page 2

## References

[1] Ferreres AR (2024). Ethical aspects of artificial intelligence in general surgical practice. Rev Col Bras Cir.

[2] Tai MC (2020). The impact of artificial intelligence on human society and bioethics. Tzu Chi Med J.

[3] Pope Leo XIV (2026). Encyclical Letter Magnifica Humanitas on Safeguarding the Human Person in the Time of Artificial Intelligence. Encyclical Letter Magnifica Humanitas on Safeguarding the Human Person in the Time of Artificial Intelligence.

[4] Sekalala S, Chatikobo T (2024). Colonialism in the new digital health agenda. BMJ Glob Health.

[5] Shaw D, Lorenzini G, Arbelaez Ossa L, Eckstein J, Steiner L, Elger BS (2025). When and what patients need to know about AI in clinical care. Swiss Med Wkly.

